# Magnesium Sulfate Treatment Reverses Seizure Susceptibility and Decreases Neuroinflammation in a Rat Model of Severe Preeclampsia

**DOI:** 10.1371/journal.pone.0113670

**Published:** 2014-11-19

**Authors:** Abbie Chapman Johnson, Sarah M. Tremble, Siu-Lung Chan, Janae Moseley, Babbette LaMarca, Keith J. Nagle, Marilyn J. Cipolla

**Affiliations:** 1 Department of Neurological Sciences, University of Vermont College of Medicine, Burlington, Vermont, United States of America; 2 Department of Obstetrics, Gynecology, and Reproductive Sciences, University of Vermont College of Medicine, Burlington, Vermont, United States of America; 3 Department of Pharmacology, University of Vermont College of Medicine, Burlington, Vermont, United States of America; 4 Department of Pharmacology, University of Mississippi Medical Center, Jackson, Mississippi, United States of America; University of Michigan, United States of America

## Abstract

Eclampsia, defined as unexplained seizure in a woman with preeclampsia, is a life-threatening complication of pregnancy with unclear etiology. Magnesium sulfate (MgSO_4_) is the leading eclamptic seizure prophylactic, yet its mechanism of action remains unclear. Here, we hypothesized severe preeclampsia is a state of increased seizure susceptibility due to blood-brain barrier (BBB) disruption and neuroinflammation that lowers seizure threshold. Further, MgSO_4_ decreases seizure susceptibility by protecting the BBB and preventing neuroinflammation. To model severe preeclampsia, placental ischemia (reduced uteroplacental perfusion pressure; RUPP) was combined with a high cholesterol diet (HC) to cause maternal endothelial dysfunction. RUPP+HC rats developed symptoms associated with severe preeclampsia, including hypertension, oxidative stress, endothelial dysfunction and fetal and placental growth restriction. Seizure threshold was determined by quantifying the amount of pentylenetetrazole (PTZ; mg/kg) required to elicit seizure in RUPP+HC±MgSO_4_ and compared to normal pregnant controls (n = 6/group; gestational day 20). RUPP+HC rats were more sensitive to PTZ with seizure threshold being ∼65% lower vs. control (12.4±1.7 vs. 36.7±3.9 mg/kg PTZ; p<0.05) that was reversed by MgSO_4_ (45.7±8.7 mg/kg PTZ; p<0.05 vs. RUPP+HC). BBB permeability to sodium fluorescein, measured in-vivo (n = 5–7/group), was increased in RUPP+HC vs. control rats, with more tracer passing into the brain (15.9±1.0 vs. 12.2±0.3 counts/gram ×1000; p<0.05) and was unaffected by MgSO_4_ (15.6±1.0 counts/gram ×1000; p<0.05 vs. controls). In addition, RUPP+HC rats were in a state of neuroinflammation, indicated by 35±2% of microglia being active compared to 9±2% in normal pregnancy (p<0.01; n = 3–8/group). MgSO_4_ treatment reversed neuroinflammation, reducing microglial activation to 6±2% (p<0.01 vs. RUPP+HC). Overall, RUPP+HC rats were in a state of augmented seizure susceptibility potentially due to increased BBB permeability and neuroinflammation. MgSO_4_ treatment reversed this, increasing seizure threshold and decreasing neuroinflammation, without affecting BBB permeability. Thus, reducing neuroinflammation may be one mechanism by which MgSO_4_ prevents eclampsia during severe preeclampsia.

## Introduction

Preeclampsia (PE) is a hypertensive complication of pregnancy that involves many organ systems, including the kidney, liver and brain [1]. Some of the most serious complications of PE involve neurologic symptoms and include uncontrolled vomiting, severe and persistent headache, visual disturbances, unexplained seizure (eclampsia), coma and death [1]. Eclampsia is a life-threatening condition with high maternal and fetal morbidity and mortality [2], [3]. The mechanism by which de novo seizure occurs in women with PE is not known, however, studies have shown that the brain is more excitable during pregnancy and PE, suggesting a lower seizure threshold that may contribute to de novo seizure. For example, network excitability in brain slices of pregnant mice was increased compared to virgin animals [4]. Further, a lower seizure threshold was reported in a lipopolysaccharide (LPS)-induced rat model of PE compared to normal pregnancy [5], suggesting pregnancy and PE may predispose the brain to seizure through increased neuronal excitability. However, the mechanism by which neuronal excitability is augmented in PE is unknown.

One of the primary mechanisms by which neuronal excitability can increase is through activation of microglia and neuroinflammation [6], [7]. Microglial activation under conditions of peripheral inflammation has been shown to decrease seizure threshold via a promotional effect on neuronal excitability [8]. In addition, increased blood-brain barrier (BBB) permeability can result in neuroinflammation by allowing passage of serum constituents into the brain that activate microglia [9]–[12]. A previous study showed that circulating factors present during normal pregnancy are hyperexcitable to the brain through activation of microglia, and increase network excitability in a hippocampal slice culture model [13]. In addition, circulating factors during PE have been shown to increase BBB permeability that could potentially pass into the maternal brain to promote neuroinflammation and decrease seizure threshold [14], [15]. In the present study, we hypothesized that PE produces a state of neuroinflammation that lowers seizure threshold. We further hypothesized that BBB disruption during PE leads to microglia activation and is a mechanism by which seizure threshold is lowered during PE.

Magnesium sulfate (MgSO_4_) is currently the most effective and commonly administered drug for eclamptic seizure prophylaxis and reduces the incidence of eclampsia by ∼50% [16]–[20]. MgSO_4_ is administered to women at relatively high doses to raise serum levels to between 4.2–8.4 mg/dL over 12–24 hours [16]. However, despite its apparent effectiveness, dangerous side effects are associated with MgSO_4_ use, including the potential for areflexia, respiratory paralysis and cardiac arrest [16]. Further, although it is the preferred treatment strategy in women with PE, the exact mechanism by which MgSO_4_ prevents eclampsia is not clear and may be multifaceted. Animal studies have provided evidence that MgSO_4_ has protective actions at the BBB, lowering permeability during acute hypertension, and reducing hyperosmolar-induced disruption of the BBB [21], [22]. We hypothesized that MgSO_4_ treatment during PE increases seizure threshold by preserving the integrity of the BBB and preventing microglial activation, thereby decreasing neuroinflammation.

In the present study, we developed a rat model of severe PE that incorporated placental ischemia and maternal endothelial dysfunction that are thought to contribute to the pathogenesis of PE [23]. In particular, we sought to model severe PE that encompasses both fetal and maternal symptoms and has the greatest risk of life-threatening complications [23], [24]. This model used the reduced uteroplacental perfusion pressure (RUPP) model of placental ischemia [25] combined with a high cholesterol diet previously shown to cause maternal endothelial dysfunction [15], [26]. Using this model, we investigated the effect of severe PE on seizure threshold, BBB permeability in vivo and neuroinflammation. Further, rats with severe PE were treated with a clinically relevant dose of MgSO_4_ for 24 hours, and the effect of MgSO_4_ on these parameters was also determined.

## Methods

### Animals and ethics statement

All experiments were conducted using timed-pregnant Sprague Dawley rats that were 14–16 weeks old (Charles River, Canada). All rats were used experimentally and euthanized late in gestation (day 20 of a 22 day gestation), as this is when eclampsia occurs most often [1]. Rats of the same gestational age were housed in pairs in the University of Vermont Animal Care Facility, an Association for Assessment and Accreditation of Laboratory Animal Care International accredited facility. All procedures were approved by the Institutional Animal Care and Use Committee at the University of Vermont and conducted in accordance with the National Institute of Health Guide for the Care and Use of Laboratory Animals.

### Rat model of severe PE and assessment of pregnancy outcome

To model severe PE, we combined the RUPP model of placental ischemia with a high cholesterol diet (RUPP+HC) previously shown to cause hyperlipidemia by increasing total plasma cholesterol, maternal endothelial dysfunction and increased blood pressure [15], [26]. Forty-eight pregnant rats were fed a high cholesterol diet (Prolab 3000 rat chow with 2% cholesterol and 0.5% sodium cholate; Scotts Distributing Inc., Hudson, NH) on days 7–20 of gestation. To induce placental ischemia, on day 14 of pregnancy while maintained on a high cholesterol diet, a midline abdominal incision was made under isoflurane anesthesia and a silver clip (diameter 0.203 mm) placed on the distal aorta, just proximal to the iliac bifurcation and distal to the renal and mesenteric arteries. Silver clips (diameter 0.10 mm) were placed on the arteries of the uterine arcade before the first segmental branch to the uteroplacental unit. This is an established method of reducing uteroplacental perfusion pressure by ∼40% [25]. Pregnant rats that had a sham surgery (n = 4) underwent the same surgical procedure, excluding the placement of silver clips. Animals were weighed prior to use and euthanized by decapitation after experimentation under anesthesia. Trunk blood was collected and serum stored at −80°C until use. Uterine horns were examined for total number of pups and any reabsorbed fetuses. To assess growth restriction and placental disease, pups and placenta from some RUPP+HC rats (n = 5) were removed, weighed individually and compared to control rats (n = 3).

### Measurement of conscious, unrestrained arterial blood pressure

Separate groups of normal late-pregnant (Late-Preg; n = 6) and RUPP+HC rats (n = 6) were implanted with indwelling carotid catheters on day 18 of gestation. Under isoflurane anesthesia, the left common carotid artery was exposed and cannulated with saline filled V-3 tubing (SCI) which was tunneled to the back of the neck and externalized. On day 19 of pregnancy, rats were lightly anesthetized with isoflurane (1–2% for 3–4 minutes) and a pressure transducer (BIOPAC, Inc., Goleta, CA, USA) connected to the indwelling carotid catheter. Rats were placed in a rectangular, clear plastic modular chamber (10”×7”×5”) with a metal grid floor, large enough to move freely. Average conscious, unrestrained systolic blood pressures were recorded using Acq*Knowledge* software (BIOPAC, Inc., Goleta, CA, USA). One RUPP+HC rat died due to surgical complications during catheter implantation and was excluded.

### Measurement of circulating markers of endothelial dysfunction and oxidative stress

Commercially available rat ELISA kits for endothelin-1 (ET-1; R&D Systems, Minneapolis, MN, USA) and free 8-isoprostane (Caymen Chemicals, Ann Arbor, MI, USA) were used to measure circulating factors in the serum of Late-Preg (n = 6) and RUPP+HC (n = 6–8) rats. Serum samples were diluted 1∶3 for measurements of free 8-isoprostane otherwise samples were measured undiluted. All samples were measured in duplicate.

### MgSO_4_ treatment in RUPP+HC rats

Eighteen RUPP+HC rats were injected s.c. the morning of day 19 of pregnancy with 270 mg/kg 1.0 M MgSO_4_ (RUPP+HC+MgSO_4_). Four hours later, rats were briefly anesthetized with 2% isoflurane and three 2 mL osmotic minipumps (Alzet, Cupertino, CA, USA) primed with 1.0 M MgSO_4_ were implanted s.c. between the shoulder blades. On day 20 of pregnancy, 1.5 hours prior to surgery and experimentation rats were injected with a second bolus of 270 mg/kg 1.0 M MgSO_4_ s.c. After experimentation, rats were euthanized by decapitation under anesthesia and serum collected and stored at −80°C until use. Serum [Mg^2+^] was measured using a colorimetric assay (BioVision Inc., San Francisco, CA, USA) according to the manufactures' instructions and compared to RUPP+HC rats that did not receive treatment with MgSO_4_ (n = 4/group).

### Measurement of seizure threshold, susceptibility and severity, and brain water content

Separate groups of Late-Preg (n = 10), RUPP+HC (n = 6) and RUPP+HC+MgSO_4_ (n = 6) were anesthetized initially with isoflurane (1–3% in oxygen) for intubation, electrode placement and instrumentation. Animals were mechanically ventilated to maintain blood gases and pH within normal physiological ranges. Body temperature was monitored with a rectal thermometer and maintained with a heating pad at 37°C throughout the experiment. The dorsal surface of the head was shaved to expose the scalp and silver subdermal corkscrew electrodes (Ambu, Glen Burnie, MD, USA) were implanted under the scalp and secured in place with collodion glue. Electroencephalography (EEG) was recorded unipolarly using a MP150 acquisition system (BIOPAC System Inc., Goleta, CA, USA) to monitor generalized seizure. The recording electrode was placed over the right parieto-occipital cortex (5±0.16 mm lateral and 7±0.16 mm posterior to bregma) [27], a reference electrode was placed in the soft tissue of the snout and a ground electrode placed posterior to the right ear. Signals were amplified and filtered (low frequency filter, 0.1 Hz; high frequency filter 35.0 Hz) and sampled at 1.0 kHz. After placement of electrodes, the animal was placed in supine position for placement of venous and arterial catheters. Femoral arteries were cannulated to obtain blood samples for blood gas measurements and continuous measurement of arterial blood pressure via a pressure transducer (BIOPAC Systems Inc., Goleta, CA, USA). As placement of a silver clip on the distal aorta in rats with RUPP made monitoring blood pressure in the femoral artery inaccurate, blood pressures were measured in the axillary artery, as done previously [28]. Femoral veins were cannulated for administration of the anesthetic chloral hydrate and infusion of the chemoconvulsant pentylenetetrazole (PTZ). PTZ was chosen because it reliably elicits seizure by its antagonistic action at the main inhibitory receptors in the brain, gamma-aminobutyric acid (GABA) type A receptors [29]. After instrumentation, animals were tapered off isoflurane and anesthesia maintained by continuous intravenous infusion of chloral hydrate (50 mg/mL; 30 µL/min). Chloral hydrate was used because it is thought to not depress neural function, and is the preferred anesthetic for studies measuring EEG [30], [31]. Seizure was induced by a timed infusion of PTZ (10 mg/mL; 1 mL/min) that was stopped at the first onset of spikewave discharges. Seizure threshold was calculated as the amount of PTZ (mg/kg) required to elicit electrical seizure: T_infusion_ * R_infusion_ * [PTZ]/BW where T_infusion_ is the time of infusion in min, R_infusion_ is the rate of infusion in mL/min, [PTZ] is the concentration of PTZ in mg/mL, and BW is the body weight in kg. Seizure susceptibility scores were also calculated: bw * 10/v where bw is body weight in grams and v is volume of PTZ infused in µL [8]. Baseline blood pressures were taken 30 seconds prior to PTZ infusion and at seizure onset. EEG was recorded for 30 minutes post-PTZ infusion and seizure severity assessed by counting the number of recurring seizures and calculating the percent of the post-infusion period spent in seizure. After 30 minutes animals were euthanized under chloral hydrate anesthesia by decapitation and brains immediately removed. The posterior cerebral cortex was isolated and weighed wet (weight_wet_), then dried in a laboratory oven at 90°C for 24 hours and re-weighed dry (weight_dry_). Percent water content was determined by wet:dry weights using the following formula: (weight_wet_ - weight_dry_/weight_wet_) * 100. The posterior cortex was chosen for measurements as this is a primary brain region affected in women with PE and eclampsia [32]. Four Late-Preg rats were excluded because blood gases were outside of the physiological range.

### Measurement of in vivo BBB permeability

Permeability of the BBB was measured in Late-Preg (n = 6), RUPP+HC (n = 5) and RUPP+HC+MgSO_4_ (n = 8) using previously described methods with modifications [33]. Briefly, animals were anesthetized and instrumented similarly as during seizure threshold measurements, excluding EEG electrode placement and substituting the catheter for PTZ with fluorescent tracers. While under chloral hydrate anesthesia, fluorescent tracers were infused into the femoral vein (0.5 mL/min for 2.25 min) and allowed to circulate for 10 minutes. Sodium fluorescein (0.1%; mol wt 476 Da; Stokes-Einstein radius ∼0.45 nm) and 70-kDa Texas red dextran (0.5 mg/mL; Stokes-Einstein radius ∼7.0 nm) in lactated Ringer's solution and heparin were used to distinguish size selectivity to small and large solutes, respectively. A thoracotomy was performed, a needle inserted into the left ventricle of the heart and the right atrium cut to allow for drainage of blood and tracers. Using an infusion pump, the circulation was flushed with 60 mL lactated Ringer's solution (5.0 mL/min) until the circulation was clear of blood. Animals were decapitated, the brain removed and the posterior cerebral cortex isolated and weighed. Each brain section was homogenized in 5.0 mL 0.1 M PBS, 5.0 mL 50% trichloroacetic acid (TCA) added, vortexed for 1 min, and centrifuged at 4°C for 10 min at 4500 rpm (Sorvall Legend ×1R, Thermo Scientific, Waltham, MA, USA. The supernatant was removed and re-centrifuged. The fluorescence of the supernatant was determined at excitation and emission wavelengths of 460 and 515 nm for sodium fluorescein and 595 and 615 nm for Texas red dextran. Background emissions of 50∶50 lactated Ringer's and 50% TCA were subtracted and samples normalized to brain weight to compare fluorescence as counts/gram of brain tissue. One Late-Preg rat and one RUPP+HC+MgSO_4_ rat were excluded due to surgical complications.

### Quantification and morphological assessment of microglia

Separate groups of Late-Preg (n = 8), RUPP+HC (n = 3) and RUPP+HC+MgSO_4_ (n = 4) rats were euthanized under isoflurane anesthesia and brains immediately removed. A 3 mm coronal section (4–7 mm posterior to bregma) of the posterior cerebral cortex was taken and fixed in 10% buffered formalin at 4°C overnight, then transferred to 0.1 M PBS and paraffin embedded. Immunohistochemical staining for ionized calcium-binding adapter molecule 1 (Iba1; Wako, Richmond, VA), a marker for microglia, was done using standard procedures. Briefly, tissue was sectioned at 4 µm on a Leica 2030 paraffin microtome. Slides were allowed to air dry overnight at room temperature and then baked for one hour at 60°C. Following deparaffinization and rehydration, the sections underwent antigen retrieval with DAKO Target Retrieval Solution, pH 6.0 in 50% glycerol at 95°C for 20 min. Sections were treated with 1% bovine serum albumin, 10% normal goat serum and 0.1% Triton X-100. The tissue was incubated overnight at room temperature with Iba1 antibody (1 µg/mL) and one hour in Cyanine 3 dye (1∶100). For each brain section, four micrographs of cerebral cortex were captured using an Olympus BX50 microscope at 20× magnification. Each Iba1^+^ cell was assessed by its morphology and activation state ranked using a graded scale from 1 (relatively inactive) to 4 (relatively active). Cells with highly ramified, long processes with a scattered, irregularly shaped cell body were ranked in state 1. Cells with an asymmetrical cell body and many long, defined processes were ranked in state 2. Cell bodies that were more rounded with several shorter, thicker processes were ranked 3, and large, round amoeboid-like cell bodies with few to no processes were ranked in state 4 [34]. To assess microglia, two analyses were performed. First, the percentage of cells in each activation state was calculated for each micrograph and averaged per group. Second, total number of Iba1^+^ cells were counted per mm^2^ and averaged for each group. A group of sham-operated rats were assessed for microglial activation to confirm the surgery alone did not activate microglia. Two evaluators that were blinded to group performed all morphological assessments.

### Measurement of cerebral blood flow autoregulation and brain water content

Separate groups of Preg (n = 6) and RUPP+HC (n = 10) rats were anesthetized initially with isoflurane (1–3% in oxygen) for intubation and instrumentation, after which anesthesia was maintained with a bolus intravenous injection of chloral hydrate (200 mg/kg into femoral vein). Animals were mechanically ventilated to maintain blood gases and pH within normal physiological ranges. Body temperature was monitored with a rectal thermometer and maintained with a heating pad at 37°C throughout the experiment. Relative cerebral blood flow (rCBF) was measured transcranially in the posterior cerebral cortex using laser Doppler flowmetry. The left side of the medioposterior skull was exposed and the bone thinned. A laser Doppler probe (Perimed, Ardmore, PA, USA) was affixed 2 mm lateral to the sagittal suture and 1 mm anterior to the lambdoid suture to measure rCBF in the posterior cerebral artery territory. Femoral arteries were cannulated to obtain blood samples for blood gas measurements and measurement of arterial blood pressure via a pressure transducer (Living Systems Instrumentation, Inc., Burlington, VT, USA). Femoral veins were cannulated for administration of chloral hydrate and acute infusion of phenylephrine. To measure CBF autoregulation, phenylephrine was infused intravenously at an increasing rate of 4–48 µg/min, and blood pressure and rCBF measured simultaneously, as previously described [35]. After experimentation, while under anesthesia, rats were euthanized by decapitation, brains immediately removed and percent water content measured by wet:dry weights. One RUPP+HC rat was excluded because of technical issues with placement of the laser Doppler probe. The percent increase in CBF of one RUPP+HC rat was greater than two standard deviations from the mean, and therefore excluded as a statistical outlier. Animals were not randomized due to the use of timed-pregnant rats.

### Drugs and solutions

MgSO_4_, phenylephrine, chloral hydrate, PTZ, sodium fluorescein and TCA were purchased from Sigma Aldrich (St Louis, MO, USA) and all were made daily in sterile lactated Ringer's solution except MgSO_4_, which was ready-to-use. Texas red dextran was purchased from Invitrogen (Life Technologies, Grand Island, NY, USA) and made daily in sterile lactated Ringer's solution.

### Statistical analyses

Data are presented as mean ± standard error of mean. Physiological parameters were compared between Late-Preg and RUPP+HC rats using analysis of variance (ANOVA). Pup and placental weights were compared using ANOVA with the n-value being total pups and placentas in each group. Differences in circulating levels of ET-1 and free 8-isoprostane were also compared using ANOVA. Percent change in rCBF was compared between Late-Preg and RUPP+HC rats at pressures between 100 and 180 mmHg using ANOVA. Comparisons of physiological parameters, seizure threshold, susceptibility, severity, BBB permeability, % water content and microglial activation between Late-Preg, RUPP+HC and RUPP+HC+MgSO_4_ rats were done using a one-way ANOVA with a Bonferroni's *post-hoc* test to correct for multiple comparisons. Differences were considered significant at p<0.05.

## Results

### Severe PE caused hypertension, oxidative stress, endothelial dysfunction, and fetal and placental growth restriction

Table 1 shows physiological parameters of Late-Preg and RUPP+HC rats. RUPP+HC rats weighed significantly less on day 20 of gestation compared to Late-Preg rats. This decrease in maternal body weight was not due to significant pup loss or fetal reabsorptions, as the number of pups and reabsorbed fetuses were not statistically different between groups. However, RUPP+HC caused both fetal and placental growth restriction, as shown by significant reductions in pup and placental weights. Further, rats with severe PE had increased systolic blood pressure compared to Late-Preg rats. When serum markers of oxidative stress and endothelial dysfunction were compared, severe PE was associated with significantly higher levels of circulating free 8-isoprostane compared to control rats: 369±25 pg/mL for RUPP+HC vs. 281±8 pg/mL for Late-Preg; p<0.01 and ET-1: 0.99±0.25 pg/mL for RUPP+HC vs. 0.46±0.06 pg/mL for Late-Preg; p<0.05.

**Table 1:** Physiological parameters in models of normal pregnancy (Late-Preg) and severe preeclampsia (RUPP+HC).

	Late-Preg (n)	RUPP+HC (n)
Body Weight (grams)	460.0±10.0 (3)	371.0±21.2 (5) *
# Pups	14.7±1.2 (3)	11.2±3.4 (5)
# Reabsorptions	1.7±0.9 (3)	3.0±2.5 (5)
Pup Weight (grams)	2.5±0.03 (44)	2.2±0.02 (56) **
Placental Weight (grams)	0.46±0.01 (44)	0.42±0.01 (56) **
Blood Pressure (mmHg)	114±1 (6)	138±3 (4) **

* p<0.05; ** p<0.01 vs. Late-Preg by ANOVA.

### Severe PE was associated with decreased seizure threshold and increased seizure susceptibility

**Figure 1:**
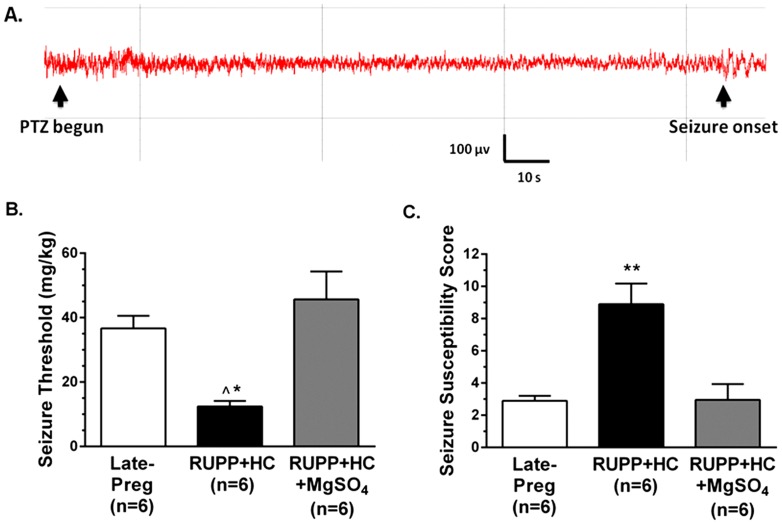
Effect of severe preeclampsia and magnesium sulfate (MgSO_4_) treatment on seizure threshold and susceptibility. (A) Representative EEG tracing during timed-infusion of pentylenetetrazole (PTZ). Black arrows indicate when PTZ infusion begun and the onset of spike-wave discharges, or seizure onset. (B) Seizure threshold was significantly lower in rats with severe preeclampsia (RUPP+HC), and treatment with MgSO_4_ (RUPP+HC+MgSO_4_) significantly increased seizure threshold back to late-pregnant (Late-Preg) control levels. (C) Rats with severe PE had significantly higher seizure susceptibility scores compared to Late-Preg controls that were reversed in RUPP+HC+MgSO_4_ rats. * p<0.05 vs. Late-Preg; **∧** p<0.01 vs. RUPP+HC+MgSO_4_; ** p<0.01 vs. all groups by one-way ANOVA with *post-hoc* Bonferroni test.

To determine if severe PE was a state associated with increased neuronal excitability, seizure threshold was measured in vivo in RUPP+HC and Late-Preg rats by timed infusion of PTZ and simultaneous EEG recording. Figure 1A shows a representative EEG tracing in a Late-Preg rat during PTZ infusion that had spike wave discharges indicative of seizure onset after 2.4 min of infusion. Seizure threshold was significantly lower in RUPP+HC rats, requiring less PTZ to elicit electrical seizure than in Late-Preg controls (Figure 1B). Further, severe PE was associated with a higher seizure susceptibility score than in Late-Preg controls, suggesting that RUPP+HC rats are in a state of increased neuronal excitability (Figure 1C). Comparison of recurring seizures and percentage of time spent seizing revealed no differences between RUPP+HC and Late-Preg rats (Table 2), indicating that although rats with severe PE were more susceptible to seizure than controls, seizure severity was similar between these groups.

**Table 2:** Assessment of seizure severity in late-pregnant (Late-Preg) rats, rats with severe preeclampsia (RUPP+HC), and severe preeclamptic rats treated with magnesium sulfate (RUPP+HC+MgSO_4_).

	# Recurrent Seizures	% of Time in Seizure
Late-Preg (n = 6)	12±3	85±7
RUPP+HC (n = 6)	13±4	86±5
RUPP+HC+MgSO_4_ (n = 6)	6±1	88±5

### MgSO_4_ treatment reversed seizure susceptibility in rats with severe PE

Treatment of RUPP+HC rats with MgSO_4_ raised serum Mg^2+^ levels into the target therapeutic range that was significantly higher than RUPP+HC rats that did not receive treatment (5.2±0.5 mg/dL for treated vs. 1.2±0.1 mg/dL for untreated; p<0.01). MgSO_4_ treatment did not affect physiological parameters or pregnancy outcome, as maternal body weight (389.2±8.9 grams; p>0.05 vs. untreated), number of pups (11.5±1.2; p>0.05) and fetal reabsorptions (1.8±0.9; p>0.05) were similar to RUPP+HC rats (see Table 1). Severe PE rats that received MgSO_4_ had significantly higher seizure threshold compared to rats with severe PE that did not receive treatment (Figure 1B). Further, MgSO_4_ reversed the increase in seizure susceptibility, lowering susceptibility scores back to control levels (Figure 1C). Despite these apparent protective effects, MgSO_4_ did not affect seizure severity, as there were no changes in either number of recurrent seizures or percent of time spent seizing with MgSO_4_ treatment (Table 2). There were no differences in any physiological parameters under anesthesia during seizure threshold measurements between groups (Table 3).

**Table 3:** Physiological parameters of late-pregnant (Late-Preg) rats, rats with severe preeclampsia (RUPP+HC), and severe preeclamptic rats treated with magnesium sulfate (RUPP+HC+MgSO_4_) under chloral hydrate anesthesia for seizure threshold measurements.

	Late-Preg (n = 6)	RUPP+HC (n = 6)	RUPP+HC+MgSO_4_ (n = 6)
Body Temp (°C)	36.8±0.2	36.4±0.1	36.5±0.1
Arterial P_O2_ (mmHg)	111±7	121±6	116±10
Arterial P_CO2_ (mmHg)	42.8±1.7	41.5±1.5	44.8±3.2

### Severe PE rats with and without MgSO_4_ had decreased seizure-induced cerebral vasogenic edema formation

**Figure 2:**
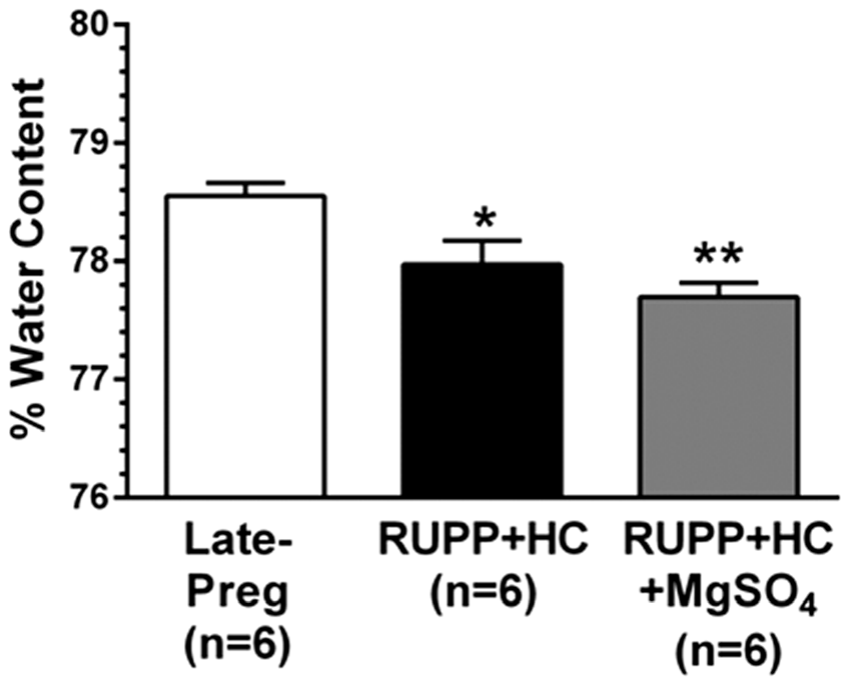
Effect of severe preeclampsia and magnesium sulfate (MgSO_4_) on seizure-induced vasogenic edema formation. Percent water content of the posterior cerebral cortex was significantly lower after seizure in rats with severe preeclampsia (RUPP+HC) compared to late-pregnant (Late-Preg) control rats. Treatment of severe preeclamptic rats with MgSO_4_ (RUPP+HC+MgSO_4_) did not affect brain water content after seizure. * p<0.05; ** p<0.01 vs. Late-Preg by one-way ANOVA with *post-hoc* Bonferroni test.

Cerebral vasogenic edema is present in ∼90% of women with eclampsia [36]. Further, the maternal brain has been shown to be more susceptible to vasogenic edema formation than the nonpregnant state under pathologic conditions including acute hypertension and in response to seizure [37], [38]. However, whether the brain during severe PE is more susceptible to seizure-induced vasogenic edema has yet to be investigated, and the effect of MgSO_4_ treatment under such conditions remains unknown. In the current study, seizure-induced vasogenic edema formation was significantly lower in RUPP+HC rats compared to Late-Preg controls, and remained unaffected by MgSO_4_ treatment (Figure 2).

### Severe PE increased BBB permeability in vivo that was unaffected by MgSO_4_ treatment

**Figure 3:**
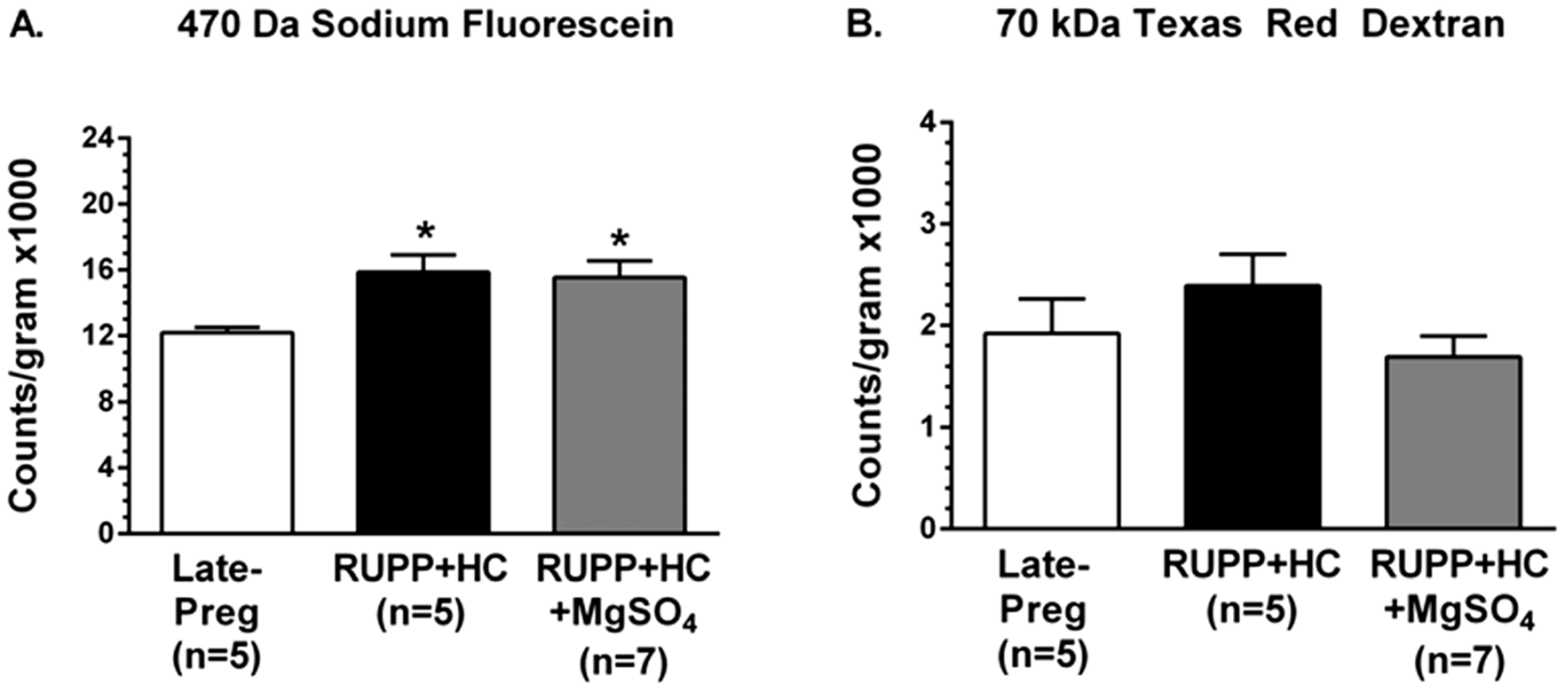
Effect of severe preeclampsia and magnesium sulfate (MgSO_4_) on in vivo blood-brain barrier (BBB) permeability to different sized solutes. (A) BBB permeability to sodium fluorescein was increased in rats with severe preeclampsia (RUPP+HC) in the posterior cerebral cortex compared to late-pregnant (Late-Preg) control rats. MgSO_4_ treatment in rats with severe preeclampsia (RUPP+HC+MgSO_4_) had no effect on BBB permeability to sodium fluorescein. (B) Permeability of the BBB to 70 kDa Texas red dextran was similar between Late-Preg and RUPP+HC rats with and without MgSO_4_ treatment. * p<0.05 vs. Late-Preg by one-way ANOVA with *post-hoc* Bonferroni test.

BBB permeability to sodium fluorescein, the small ∼470 Da solute, was increased in RUPP+HC rats compared to Late-Preg rats, with significantly more tracer passing from the cerebral circulation into the brain parenchyma in rats with severe PE (Figure 3A). There was no difference in permeability of the BBB to Texas red dextran, a larger 70 kDa tracer, between Late-Preg and RUPP+HC rats (Figure 3B). MgSO_4_ treatment administered to rats with severe PE did not affect BBB permeability, as permeability to sodium fluorescein remained higher in RUPP+HC+MgSO_4_ rats than Late-Preg controls (Figure 3A), with no change in permeability to Texas red (Figure 3B).

### Severe PE increased neuroinflammation that was reversed by MgSO_4_ treatment

**Figure 4:**
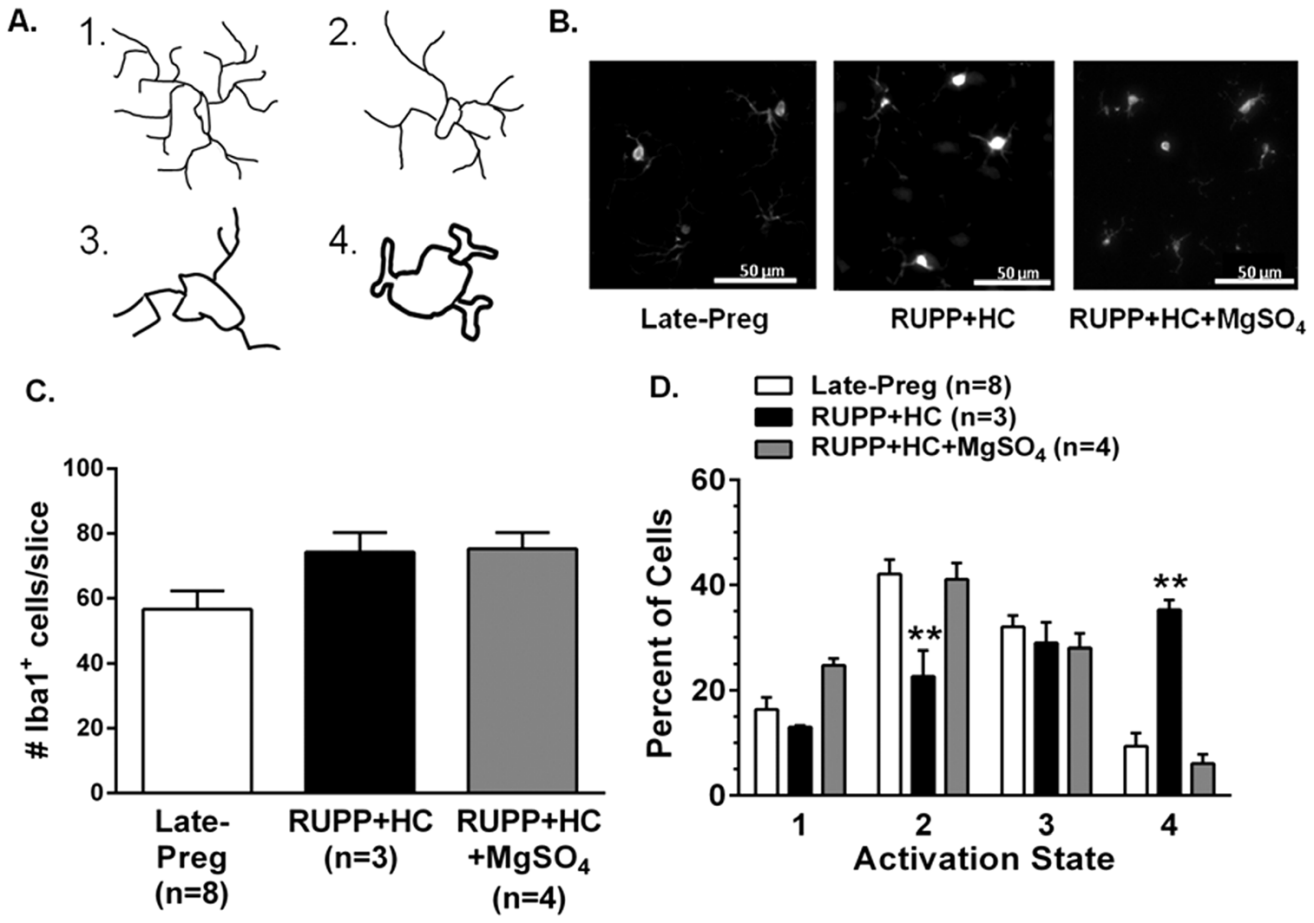
Effect of severe preeclampsia and magnesium sulfate (MgSO_4_) treatment on microglial activation. (A) Illustration of morphological changes occurring as microglia progress from their inactive state 1, marked by long ramified processes to their active state 4, indicated by a large, amoeboid-like shape. (B) Representative photomicrographs of microglial cells stained for ionized calcium-binding adapter molecule 1 (Iba1) in the posterior cerebral cortices of late-pregnant (Late-Preg) rats, rats with severe preeclampsia (RUPP+HC) and rats with severe preeclampsia treated with MgSO_4_ (RUPP+HC+MgSO_4_). (C) The number of Iba1^+^ microglia was similar between groups. (D) The percentage of microglia in active state 4 was significantly higher in RUPP+HC rats compared to Late-Preg controls. Treatment of RUPP+HC rats with MgSO_4_ decreased the percentage of cells that were active and was similar to Late-Preg controls. ** p<0.01 vs. Late-Preg and RUPP+HC+MgSO_4_ by one-way ANOVA and *post-hoc* Bonferroni test.

Figure 4A illustrates the dynamic morphological changes that occur as quiescent microglia transition to an activated state and was used as a graded scale to assess microglial activation. Figure 4B shows representative photomicrographs of Iba1^+^ microglia from the posterior cerebral cortex of Late-Preg, RUPP+HC and RUPP+HC+MgSO_4_ rats. The total number of microglia was modestly increased in rats with severe PE regardless of MgSO_4_ treatment, but was not statistically different than Late-Preg controls (Figure 4C). There was a significantly higher percent of cells in fully activated state 4 in rats with severe PE compared to controls, that was coupled by a reduction in the percent of cells in activation state 2 (Figure 4D). These findings indicate the presence of neuroinflammation in rats with severe PE. Treatment of RUPP+HC rats with MgSO_4_ decreased the percent of activated microglia, shifting back to a relatively quiescent and inactive pattern similar to Late-Preg controls, with the majority of microglial cells residing in a relatively inactive state. Sham operated rats had similar microglial activation as Late-Preg control rats (data not shown).

### CBF autoregulation and brain water content in Severe PE

**Figure 5:**
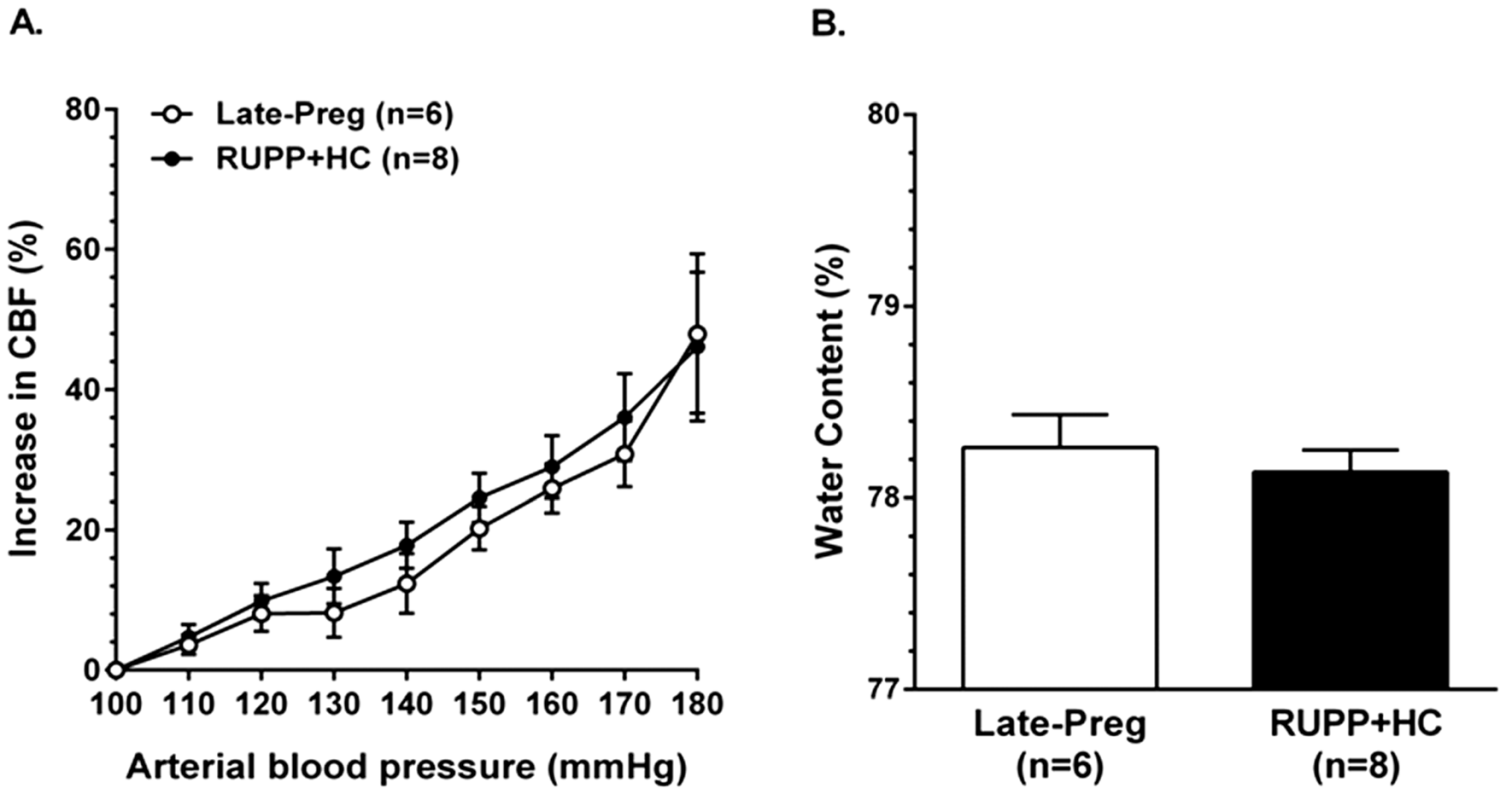
Effect of severe preeclampsia on cerebral blood flow (CBF) autoregulation and vasogenic edema formation. (A) Relative CBF (rCBF) increased similarly between late-pregnant (Late-Preg) and rats with severe preeclampsia (RUPP+HC) as arterial blood pressure was increased between 100 mmHg to 180 mmHg demonstrating intact autoregulation of CBF that was not different between groups. (B) Percent water content of the posterior cerebral cortex after CBF autoregulation measurements was similar between Late-Preg and RUPP+HC rats.

An underlying feature of neurological complications during severe PE is thought to be impairment of CBF autoregulation [39]. To compare CBF autoregulation between Late-Preg and RUPP+HC rats, rCBF vs. pressure curves were generated. Figure 5A shows that as arterial blood pressure increased, the change in rCBF was similar between Late-Preg rats and rats with severe PE and stayed below 20% until 150 mmHg (Figure 5A). To investigate if RUPP+HC rats were more susceptible than Late-Preg rats to hypertension-induced cerebral edema, percent water content of the brain was measured after measurement of CBF autoregulation. There was no difference in brain water content after phenylephrine-induced hypertension between Late-Preg and RUPP+HC rats (Figure 5B).

## Discussion

When eclamptic seizure occurs in women with early-onset severe PE there is approximately a 50% maternal mortality rate [3]. This suggests that the maternal brain is adversely affected in severe PE and at a greater risk of damage. The mechanism(s) by which eclampsia occurs is not clearly understood, but may involve a pathologic process involving BBB dysfunction, neuroinflammation and hyperexcitability of the brain. Here, we modeled severe PE by introducing placental ischemia in a setting of maternal endothelial dysfunction due to hyperlipidemia. RUPP+HC rats had increased blood pressure and were in a state of oxidative stress and endothelial dysfunction, as indicated by elevated circulating levels of free 8-isoprostane and ET-1. Further, rats with severe PE had fetal and placental growth restriction that was not associated with a change in number of pups or fetal reabsorptions. This is similar to women with early-onset severe PE that have intrauterine growth restriction and placental disease [40]. Importantly, rats with severe PE were in a state of increased seizure susceptibility, with seizure threshold being significantly lower than normal pregnant controls. Seizure susceptibility in rats with severe PE was associated with increased BBB permeability to small solutes and microglial activation. However, there was no change in seizure severity between Late-Preg and RUPP+HC rats, indicating that regulatory mechanisms limiting prolonged seizure activity were similar to the control state, despite neuroinflammation in rats with severe PE. Thus, the brain seems to be at a greater risk of seizure due to breakdown of the BBB and associated neuroinflammation in this rat model of severe PE.

Neuroinflammation, demonstrated in the current study by activated microglia, can increase neuronal excitability through microglial secretion of pro-inflammatory cytokines such as tumor necrosis factor alpha (TNFα) [8], [13]. TNFα causes trafficking of excitatory receptors to the neuronal cell surface and simultaneous internalization of inhibitory receptors, resulting in a net increase in neuronal excitability [8], [41], [42]. Thus, it is possible that in this model of severe PE, lower seizure threshold and increased seizure susceptibility were a consequence of neuroinflammation because they also had marked microglial activation. In the current study, severe PE rats treated with MgSO_4_ had significantly less microglial activation that correlated with increased seizure threshold. In fact, MgSO_4_ treatment returned neuroinflammation and seizure susceptibility to the level of normal pregnant controls. However, the effect of MgSO_4_ appeared to be a direct effect on microglial activation, as opposed to limiting BBB permeability. MgSO_4_ has been shown to limit LPS-induced microglial secretion of pro-inflammatory cytokines in cell culture through inhibition of L-type calcium channels and subsequent reduction in downstream signaling of nuclear factor kappa B (NF-κB), a transcription factor involved in inflammatory pathways [43]–[45]. However, to our knowledge, this is the first study showing that MgSO_4_ treatment increases seizure threshold in severe PE via a quiescent effect on activated microglia in vivo. This is in agreement with a recent study showing MgSO_4_ treatment increased seizure threshold in a LPS model of PE, however, the mechanism by which this occurred was not investigated [5].

Overall the effect of MgSO_4_ treatment on seizure threshold appeared to be due to reducing neuroinflammation, and not a protective effect at the BBB. In the present study, rats with severe PE had selectively increased BBB permeability to small, but not large solutes. Increased permeability to sodium fluorescein but not 70 kDa Texas red dextran suggests size-selectivity of the increased permeability and indicates modest tight junction disruption [46], [47]. However, as MgSO_4_ is thought to decrease BBB permeability through a calcium-antagonistic action on tight junction permeability [16], [21], [22], [48], it was surprising that there was no effect of MgSO_4_ on BBB permeability in rats with severe PE. Instead, the increase in BBB permeability to sodium fluorescein may indicate an increase in transcellular permeability that may be calcium-independent, which could further explain the lack of effect of MgSO_4_ treatment on severe PE-associated BBB permeability.

The lack of increase in permeability to large solutes is in contrast to two recent studies using the RUPP model without high cholesterol treatment. Both studies report increased BBB permeability to Evan's Blue in RUPP compared to pregnant control rats [49], [50]. One explanation of these contrasting results is the different methodology used. Evan's Blue binds to albumin that changes during pregnancy and PE [51]–[54]. Thus, the use of 70 kDa Texas red dextran that does not bind to albumin likely provides more reliable results. Further, the two studies reporting increased BBB permeability to Evan's Blue in RUPP rats allowed the tracer to circulate for a longer duration of time: 3–24 hours versus 10 minutes in the current study [49], [50]. These longer time frames likely provide more variable results due to differences in clearance by cerebrospinal fluid or other tissues, including placenta [55]–[57]. Regardless, in the present study, RUPP+HC rats had selectively increased BBB permeability that may contribute to lowering seizure threshold by allowing passage of serum constituents into the brain that activated microglia and created a neuroinflammatory state.

Cerebral edema formation is considered a leading cause of the neurological symptoms that occur in severe PE, including eclamptic seizure [58]–[60]. In fact, approximately 90% of women with eclampsia have vasogenic cerebral edema formation as indicated by diffusion-weighted MRI [36]. Seizure itself leads to BBB disruption, making it difficult to determine if edema is the cause of or a consequence of eclamptic seizure [10], [61]. A recent study using PTZ to induce seizure in normal pregnant rats found that the maternal brain was more susceptible to seizure-induced vasogenic edema formation than the nonpregnant state [38]. This may be an effect of increased plasma volume during pregnancy that, under conditions of BBB disruption such as seizure, may drive water into the brain. In the current study, however, brain water content after seizure was significantly lower in RUPP+HC rats compared to Late-Preg rats, with no effect of MgSO_4_ treatment. Plasma volume contraction is known to occur in women with PE that may underlie the decreased vasogenic edema formation in severe PE rats [62]. This would also support the concept that increased plasma volume contributes to increased seizure-induced edema. Further, plasma volume contraction could also explain the significant reduction in maternal body weight that occurred in rats with severe PE. Although the brain does not seem to be more susceptible to seizure-induced vasogenic edema formation in severe PE, the cerebral circulation appears to be compromised, indicated by increased BBB permeability and neuroinflammation that could increase the risk of brain injury during eclampsia.

The cerebral circulation is thought to have a central role in neurologic complications associated with PE [3]. In fact, cerebrovascular events such as edema and hemorrhage account for ∼40% of maternal deaths [3]. Development of neurologic symptoms in women with PE is thought to involve the impairment of CBF autoregulation that is associated with decreased cerebrovascular resistance, hyperperfusion of the brain and vasogenic edema formation [32], [39]. Studies that have assessed dynamic CBF autoregulation in women with PE using transcranial Doppler (TCD) to measure changes in CBF velocity in the middle cerebral artery in response to hemodynamic fluctuations have found that CBF autoregulation appears to be intact in PE [39], [63], [64]. The findings in the current study showing intact CBF autoregulation in rats with severe PE supports these previous findings in humans. Further, as autoregulation remained intact in RUPP+HC rats, it was not surprising that there was no difference in brain water content after autoregulation measurements between groups. However, a recent study investigating CBF autoregulation in rats with RUPP without a high cholesterol diet report that autoregulation was impaired [50]. While it is unlikely that the addition of a high cholesterol diet in the current study restored autoregulation, it is more likely that methodological differences account for this discrepancy. Specifically, the current study assessed CBF autoregulation in the posterior cerebral cortex where as Warrington et al. investigated autoregulation in the anterior brain region [50]. Thus, it is possible that regional differences exist due to the primary blood supply differing between regions that may explain the conflicting findings between these two studies. Further, in the current study all catheters for continuous arterial blood pressure measurement and drug delivery were placed in distal systemic arteries such as the femoral arteries/veins to avoid disrupting blood flow to the brain. Warrington et al. used a carotid catheter to continuously monitor blood pressure while simultaneously recording CBF and cannulated one jugular vein for drug infusion [50]. As the carotid arteries and jugular veins are important for hemodynamics and CBF, their cannulation may account for the discrepancies in the effectiveness of CBF autoregulation between these studies. Regardless, the current study suggests that CBF autoregulation in the posterior cerebral cortex in rats with severe PE is similar to Late-Preg control rats. Thus it is not likely that impaired CBF autoregulation contributes to decreased seizure threshold in rats with severe PE.

In summary, PE is a heterogeneous disorder that seems to manifest along a spectrum of severity and symptoms [1], [65]. It remains unclear why some women with PE develop neurologic symptoms such as seizure, and others do not. That eclamptic seizure is more often fatal in women with severe PE stresses the importance of gaining a clear understanding of the disease processes leading to seizure onset. This study provides insight into the etiology of eclamptic seizure in severe PE, as it seems to involve a pathologic process of compromised BBB function and subsequent neuroinflammation, resulting in increased seizure susceptibility. By reducing neuroinflammation, MgSO_4_ effectively increased seizure threshold, without affecting BBB permeability. Overall, preventing eclampsia, particularly during severe PE, is important in preventing maternal and fetal morbidity and mortality worldwide. Further, understanding the mechanism by which MgSO_4_ functions as a seizure prophylactic in PE may lead to more targeted therapies and avoid unnecessary risks associated with MgSO_4_ treatment.
